# Using Biology to Create Complex Patterns

**DOI:** 10.1371/journal.pbio.0020448

**Published:** 2004-12-07

In his seminal exploration of the properties of living organisms, *What Is Life?*, Erwin Schroedinger concluded that life depends in large part on storing and processing information. For genetic material to carry the diverse instructions required for living processes, he proposed, it must be stored in an aperiodic crystal. Just nine years later, it was clear that DNA is indeed an aperiodic crystal and that genetic information is conveyed through this irregular pattern. Much like computers, biological systems are programmed to follow a precise set of rules, or algorithms, to store information and solve problems. These biological algorithms direct all manner of biochemical processes to create complex patterns and structures by chemically modifying and assembling individual components.

Of course, cells use biochemical circuits not electronic circuits. Single tubulin proteins, for example, follow precise rules of chemistry and physics to spontaneously self-assemble, or polymerize, into the microtubules essential for cell transport and motility. The proteins' binding interactions effect rules that specify how the pieces fit together to form the resulting structure. They also specify when and how tubulins assemble from a nucleation complex—a molecular algorithm governing the logic of polymerization. These complex structures self-assemble with remarkably few mistakes. Though considered quite simple, little is understood about the principles that govern programmable structural order underlying this type of spontaneous self-assembly.

In crystals, the simplest example of spontaneous self-assembly, subunits of the whole are arranged in a repeating pattern that extends indefinitely in all directions. If you know the position of one unit in the pattern, you can tell the exact position of every other unit. In a new study, Rothemund and colleagues use DNA to show that crystal growth can be programmed to create specific aperiodic patterns. Inspired by a model of crystal growth as a computational process, they have programmed DNA molecules to act as molecular building blocks, arranging themselves according to local rules that in turn create a complex global pattern. The resulting two-dimensional structures, which self-assemble from knotted DNA complexes (called tiles), grow to create a fractal pattern known as a Sierpinski triangle. These DNA structures—neither periodic (as in quartz), nor random (as in glass), nor pseudorandom (as in quasicrystals with “forbidden” five-fold symmetries)—demonstrate a form of self organization in crystalline materials determined by programmable growth rules, and are hence dubbed “algorithmic crystals.”

How can such growth algorithms be encoded in biological molecules? The rules of chemical base-pairing follow regular, predictable patterns, allowing the authors to use DNA to determine the tiles' binding interactions.[Fig pbio-0020448-g001]

**Figure pbio-0020448-g001:**
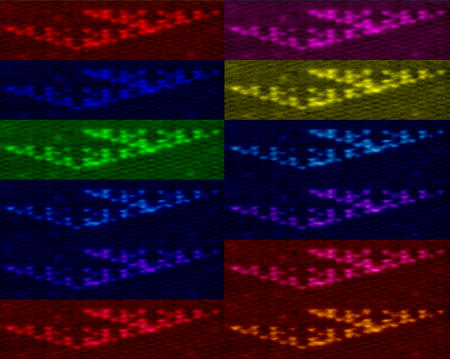
Fractal patterns from DNA

Desired binding interactions between tiles were programmed by endowing each tile with single-stranded “sticky ends” whose sequence was complementary to the sticky ends of tiles it should stick to. Each tile was either white (0) or black (1): a black tile can fit at any site where the two neighboring tiles are opposite colors, while a white tile can fit at any site where the two neighboring tiles are the same color. Logically, the new tile's color is the exclusive-or (XOR) of the tiles in the previous layer.

That such logical layer-by-layer iteration of XOR computations will produce the Sierpinski triangle is well known. What's remarkable is that DNA molecules can be programmed to grow according to this logic. With this programmable algorithmic crystal, Rothemund and colleagues demonstrate a method for designing DNA molecules capable of implementing any pattern of abstract logical tiles. What's more, the authors argue, any algorithmic crystal growth process can, in principle, be experimentally investigated using DNA self-assembly.

So how is algorithmic self-assembly related to biology? Like the algorithmic crystals, many of the self-assembled structures in biology are ordered but aperiodic. The hope is that the theoretical insights of computer science—well-honed for describing, analyzing, and programming computational systems—can direct investigations of biochemical self-assembly and information processing. And with a method for demonstrating how simple chemical and physical elements can create complex organization, Rothemund and colleagues have added a concrete experimental framework to bolster that work. (For more on DNA and complexity, see “The Emergence of Complexity: Lessons from DNA” by Chengde Mao.)

